# Supplementary material for: Investigating the relationship between teacher efficacy, job satisfaction, and digital resource utilization in assessment practices: Insights from PISA 2018 and 2022
Source: PLoS One. 2026 Jan 20;21(1):e0339475. doi: 10.1371/journal.pone.0339475 (PMC12818741; doi:10.1371/journal.pone.0339475)
Supplement: Attachment — Submitted filename: xxReview Document.docx [file pone.0339475.s001.docx]

**Review Document:**

Investigating the Relationship Between Teacher Efficacy, Job Satisfaction, and Digital Resource Utilization in Assessment Practices: Insights from PISA 2019 and 2022

**General Comments:**

Thank you very much for giving me an opportunity to review this timely and relevant article. The topic is significant, especially in the context of evolving digital education practices and teacher performance. However, the manuscript would benefit from further refinement and alignment with academic publishing standards. Below are my specific comments and suggestions:

**Formatting and Style:**

Please ensure consistency with the journal’s formatting guidelines, particularly regarding line spacing, font style, and heading levels. Review both in-text citations and reference formatting to ensure compliance with the guidelines.

**Abstract:**

A clear background and problem statement are missing. This makes it difficult to understand the study’s rationale from the outset.

**Introduction:**

The first paragraph includes too many ideas. It is recommended to narrow the focus specifically on digital education and digital resource utilization before expanding to other related themes.

While the research gap is noted, the problem statement lacks clarity and should be explicitly presented.

The theoretical framework is not well integrated. The manuscript should explain how each variable (teacher efficacy, job satisfaction, and digital resource utilization) connects with the theoretical underpinnings guiding the study.

There are too many research questions, many of which resemble qualitative inquiries. Consider consolidating them into fewer, well-structured questions that encompass the key dimensions.

Brief contextual background on the issues raised in the research questions would help establish their relevance.

**Literature Review:**

The section is well-researched, but it remains mostly descriptive. A more analytical synthesis would enhance its scholarly depth. The context specific reviews are also missed.

 While a conceptual model is included, the hypotheses are not clearly articulated in the text.

The theoretical argument supporting the relationships among variables is missing. This needs to be strengthened to frame the study appropriately.

**Methodology:**

The use of secondary data (PISA) is appropriate, but ethical considerations (e.g., data use permissions, IRB exemption) should still be acknowledged.

The SEM framework is discussed in the methodology section, including model values. It would be more appropriate to present these results in the findings section for better logical flow.

**Results:**

The presentation of results is heavily descriptive. Consider providing more critical interpretation of key findings.

The SEM model fit indices are not discussed adequately. This is essential for assessing the robustness of the model.

**Discussion:**

The theoretical engagement is limited. There is a need for deeper reflection on how the findings support or challenge existing theories.

The discussion is more narrative than analytical. Consider exploring why certain results emerged and how they compare with prior research.

The manuscript does not address alternative theoretical frameworks, such as the Technology Acceptance Model or Self-Determination Theory, which could offer valuable insights.

**Conclusion and Implications:**

The conclusion mainly repeats the findings. It should instead focus on summarizing key insights and their broader relevance.

Policy and practical implications should be clearly outlined, especially recommendations for governments, schools, and educational policymakers.

The manuscript omits a discussion of limitations, particularly methodological constraints, which are crucial for academic transparency and future research direction.

**Final Suggestions:**

A thorough language and grammar edit will improve the readability of the manuscript.

 Please consider reorganizing certain sections to improve logical progression and argumentation flow.

Overall, the study addresses an important and timely topic. With substantial revisions, particularly in theoretical alignment, methodological clarity, and analytical depth, the manuscript can make a strong contribution to the literature on digital education and teacher performance.
