# Supplementary material for: Investigating the relationship between teacher efficacy, job satisfaction, and digital resource utilization in assessment practices: Insights from PISA 2018 and 2022
Source: PLoS One. 2026 Jan 20;21(1):e0339475. doi: 10.1371/journal.pone.0339475 (PMC12818741; doi:10.1371/journal.pone.0339475)
Supplement: Attachment — Submitted filename: Reviewer_kpg.docx [file pone.0339475.s003.docx]

REVIEWER REPORT

**Reviewer**: Kaji Prasad Ghimire (K P Ghimire), Ph D

**Manuscript Title**:

Investigating the Relationship Between Teacher Efficacy, Job Satisfaction, and Digital Resource Utilization in Assessment Practices: Insights from PISA 2018 and 2022Decision:

**Authors:**

1. Dirgha Raj Joshi, Ph.D.
2. Jeevan Khanal, Ph.D.
3. Bishnu Maya Joshi, Master

**Corresponding Author:**

Jeevan Khanal, Ph.D.

Nepal Open University

Lalitpur, NEPAL

**Summary of the Manuscript**

This study looks at how three things affect the way teacher efficacy (TE), Job satisfaction (JS), and technology adoption (UIT/UDT) effect the assessment practices (AP). It uses PISA data from 24 countries in 2018 and 2022, and is based on Bandura’s Cognitive Theory. The research uses two advanced statistical methods (SEM and machine learning) to compare the differences between LMICs, UMICs, and HICs countries.

**Major Comments**

1. Literature review is long.

2. Rational of Machine Learning.

**Minor Comments**

1. Long sentences

2. Re-check UIT item code in Table 1 (repeated or miswritten (e.g., TC169Q05HA twice)

3. Language refinement

4. Acronyms on first use

5. Format equations/tables consistently

**Strengths**

1. Large, globally relevant dataset
2. Rigorous analytic approach
3. Good theoretical grounding
4. Strong construct operationalization
5. Insightful cross-economic comparisons
6. Valuable policy implications

**Overall Recommendation**

Suitable for publication

**Note:** Let me suggest using simpler words for the list below

surge, agentive, nuance, circumvent, impede
